# Reliability analysis of exonic-breakpoint fusions identified by DNA sequencing for predicting the efficacy of targeted therapy in non-small cell lung cancer

**DOI:** 10.1186/s12916-022-02362-9

**Published:** 2022-05-10

**Authors:** Weihua Li, Rui Wan, Lei Guo, Geyun Chang, Dong Jiang, Lin Meng, Jianming Ying

**Affiliations:** 1 Department of Pathology, National Cancer Center/National Clinical Research Center for Cancer/Cancer Hospital, Chinese Academy of Medical Sciences and Peking Union Medical College, No.17 Panjiayuan Nanli, Beijing, 100021 China; 2 Department of Medical Oncology, National Cancer Center/National Clinical Research Center for Cancer/Cancer Hospital, Chinese Academy of Medical Sciences and Peking Union Medical College, Beijing, China; 3 Beijing Novogene Bioinformatics Technology Co., Ltd., Beijing, China

**Keywords:** Non-small cell lung cancer, DNA sequencing, Exonic-breakpoint fusion, Alternative splicing, Targeted therapy

## Abstract

**Background:**

Diverse genomic breakpoints of fusions that localize to intronic, exonic, or intergenic regions have been identified by DNA next-generation sequencing (NGS), but the role of exonic breakpoints remains elusive. We investigated whether exonic-breakpoint fusions could predict matched targeted therapy efficacy in non-small cell lung cancer (NSCLC).

**Methods:**

NSCLC samples were analyzed by DNA NGS, RNA NGS, immunohistochemistry (IHC), and fluorescence in situ hybridization.

**Results:**

Using DNA NGS, kinase fusions were identified in 685 of 7148 (9.6%) NSCLCs, with 74 harboring exonic-breakpoint fusions, mostly anaplastic lymphoma kinase (*ALK*) fusions. RNA NGS and IHC revealed that 11 of 55 (20%) exonic-breakpoint fusions generated no aberrant transcript/protein, possibly due to open reading frame disruption or different gene transcriptional orientations. Four cases of genomic-positive but RNA/protein-negative fusions were treated with matched targeted therapy, but progressive disease developed within 2 months. Nevertheless, 44 of 55 (80%) exonic-breakpoint fusions produced chimeric transcripts/proteins, possibly owing to various alternative splicing patterns, including exon skipping, alternative splice site selection, and intron retention. Most of these genomic- and RNA/protein-positive fusion cases showed a clinical response to matched targeted therapy. Particularly, there were no differences in objective response rate (*P* = 0.714) or median progression-free survival (*P* = 0.500) between intronic-breakpoint (*n* = 56) and exonic-breakpoint *ALK* fusion subtypes (*n* = 11) among ALK RNA/protein-validated patients who received first-line crizotinib.

**Conclusions:**

Exonic-breakpoint fusions may generate in-frame fusion transcripts/proteins or not, and thus are unreliable for predicting the efficacy of targeted therapy, which highlights the necessity of implementing RNA or protein assays for functional validation in exonic-breakpoint fusion cases.

**Supplementary Information:**

The online version contains supplementary material available at 10.1186/s12916-022-02362-9.

## Background

Targeted therapies, many of which target receptor tyrosine kinases, have been extensively developed to treat patients with non-small cell lung cancer (NSCLC), leading to dramatic improvements in patient survival. Gene fusions are potent targets of therapy [1, 2]. Some kinase fusions, such as anaplastic lymphoma kinase (*ALK*), v-ros UR2 sarcoma virus oncogene homolog 1 (*ROS1*), and rearranged during transfection (*RET*), have been well characterized in NSCLC, and tyrosine kinase inhibitors (TKIs) have become a standard treatment option for advanced NSCLC patients harboring these fusions [3–6]. Other actionable kinase fusions, including neuregulin 1 (*NRG1*), neurotrophic receptor tyrosine kinases (*NTRKs*), fibroblast growth factor receptors (*FGFRs*), epidermal growth factor receptor (*EGFR*), and mesenchymal-epithelial transition factor (*MET*), are present in a diverse fraction of NSCLCs that are increasingly gaining attention with regard to novel targeted therapy development [7].

Several different methods have been developed for detecting these gene fusions in NSCLC. Among them, genomic DNA-based next-generation sequencing (DNA NGS) has been widely applied in recent years, as it enables comprehensive discovery of various actionable alterations (including mutations, fusions, and copy number variants) in NSCLC [8]. Using DNA NGS, we previously identified diverse genomic breakpoints that occur in intronic, exonic, or intergenic regions in NSCLC [9, 10]. In general, the genomic breakpoints occur at two genetic elements and form aberrant fusions that would produce typically deleterious chimeric fusion transcripts/proteins [11]. Actually, intronic-breakpoint fusions in which both genomic breakpoints localize to intronic regions usually lead to in-frame chimeric fusion transcripts/proteins because the coding sequences are completely preserved [12]. However, intergenic-breakpoint fusions, in which one or both genomic breakpoints are in intergenic regions, may or may not generate functional fusion transcripts, as revealed by RNA sequencing in our previous study and in another study [10, 13]. Fusions involving one or both exonic breakpoints (exonic-breakpoint fusions) in theory have a high chance of resulting in out-frame transcripts/proteins due to coding sequence disruption. Nonetheless, considering the potential unreliability of genomic breakpoints identified by DNA sequencing in predicting breakpoints at the transcript level [9], it is of great importance to determine whether exonic-breakpoint fusions can unequivocally generate in-frame functional fusion transcripts/proteins to accurately select NSCLCs for targeted therapy.

In this study, we systematically characterized the function of exonic-breakpoint fusions in a large number of NSCLC cases through multiplex molecular testing approaches, including DNA NGS, RNA NGS, immunohistochemistry (IHC), and fluorescence in situ hybridization (FISH). We further explored the association of exonic-breakpoint fusions with the efficacy of matched TKIs to evaluate the reliability of exonic-breakpoint fusions in predicting the benefit of targeted therapy in NSCLC.

## Methods

### Patients and tumor samples

A total of 7158 samples from 7148 patients with NSCLC who underwent molecular testing in our laboratory between January 2017 and December 2021 were retrospectively included in this study. Relevant clinical data, such as clinicopathological features and treatment history, were obtained from clinical records. The study was approved by the Institutional Review Board of the Cancer Hospital, Chinese Academy of Medical Sciences, and Peking Union Medical College. Written informed consent was obtained from each patient, and the methods were carried out in accordance with approved guidelines.

### Isolation of DNA and RNA

Tumor cellularity was evaluated through hematoxylin and eosin (HE)-stained slides by two or more pathologists, as previously described [14]. Tissues with ≥20% tumor cellularity were selected. Genomic DNA was extracted using a QIAamp DNA FFPE Tissue Kit, and RNA was extracted using a Novogene RNA Extraction Kit (Novogene, Tianjin, China). DNA and RNA quantities were measured using a Qubit 3.0 Fluorometer (Thermo Fisher Scientific, Carlsbad, CA, USA) and a NanoDrop ND-1000 Spectrophotometer (NanoDrop, Waltham, MA, USA), and the qualities were determined using an Agilent 2100 Bioanalyzer system (Agilent Technologies Inc. CA, USA).

### DNA NGS

Hybrid capture-based targeted DNA NGS was performed using a panel designed against 56 genes, as previously described [10]. Briefly, at least 50 ng of genomic DNA was used to generate sequencing libraries through sequential steps, including DNA fragmentation, PCR amplification, hybridization, and probe capture. Indexed sequencing libraries were sequenced using the Illumina Nextseq N500 platform (Illumina, San Diego, CA, USA), and sequencing data were analyzed with an in-house Molecular Diagnostics Management System to determine alterations such as mutations, copy number variants, and fusions. Fusions were identified by a variant allele frequency (VAF) ≥2% and coverage ≥1000×. All fusions were further manually analyzed by a geneticist to exclude artifactual fusion events owing to misalignment and mispriming, among other factors.

### RNA NGS

Hybrid capture-based targeted RNA NGS was performed using the TruSight RNA fusion panel (Novogene, Tianjin, China), consisting of the 95 genes listed in Additional file 1: Table S1. Briefly, 100 ng of total RNA was reverse transcribed using a random primer mix. Synthesized cDNA was end-repaired and then used to generate sequencing libraries according to the manufacturer’s instructions. The enriched libraries were sequenced with the Novaseq 6000 platform (Illumina, San Diego, CA, USA), and sequencing data were further analyzed using an in-house analysis system to identify fusion transcripts, including fusion genes and breakpoints, at the transcript level.

### IHC

ALK, ROS1, and RET expression was evaluated by an IHC assay, as previously described [10]. In brief, tissue samples were incubated with a Ventana anti-ALK (D5F3) rabbit monoclonal antibody (Ventana Benchmark XT stainer, Ventana Medical Systems Inc., AZ, USA), an anti-ROS1 (OTI1A1) mouse monoclonal antibody (Zhongshan Golden Bridge Biotechnology, Beijing, China), or an anti-RET (EPR2871) rabbit monoclonal antibody (Abcam, Cambridge, MA, USA) according to the manufacturer’s instructions. Binary scoring system (−, positive; +, negative) was used for evaluating the ALK staining results, and ALK positivity (ALK+) was identified when tumor cells showed strong granular cytoplasmic staining [15]. ROS1 and RET expression was evaluated using the following scoring scheme: -, no staining; 1+, weak staining, 2+, moderate staining, and 3+, strong staining in > 10% of tumor cells.

### FISH

FISH assays were carried out to detect *ALK* and *ROS1* fusions at the DNA level. As previously described [15], tumor tissues were sectioned, mounted onto microscope slides, and incubated with Vysis LSI ALK/ROS1 Dual color, Break Apart Rearrangement Probes (Abbott/Vysis, Abbott Park, IL, USA). The slides were evaluated under a fluorescence microscope by two expert pathologists. Positive results were identified as more than 15% of tumor cells with splitting of one or both 5′ and 3′ probe signals or with isolated 3′ probe signals.

### Efficacy evaluation of targeted therapy

The clinical responses of advanced NSCLC patients who harbored *ALK*, *ROS1*, or *RET* fusions and had received matched targeted agents were assessed by oncologists based on radiographic imaging. The optimal response, including complete response (CR), partial response (PR), stable disease (SD), and progressive disease (PD), was evaluated following the guidelines of Response Evaluation Criteria in Solid Tumors version 1.1 [16]. The cutoff date was December 31, 2021, and data were censored if patients were still alive and showed no progression at the latest follow-up date. The objective response rate (ORR) was defined as the percentage of patients with CR or PR. Progression-free survival (PFS) was calculated from the date of treatment to the date of PD or death from any cause.

### Statistical analysis

All data were analyzed using SPSS 22.0 software (Chicago, IL, USA). Differences in clinicopathologic variables between the two groups were investigated by the chi-square test or Fisher’s exact test. PFS was analyzed using the Kaplan-Meier method and log-rank test. A two-sided *P* < 0.05 was considered statistically significant.

## Results

### Characteristics of fusion-positive NSCLCs identified by DNA NGS

**Fig. 1:**
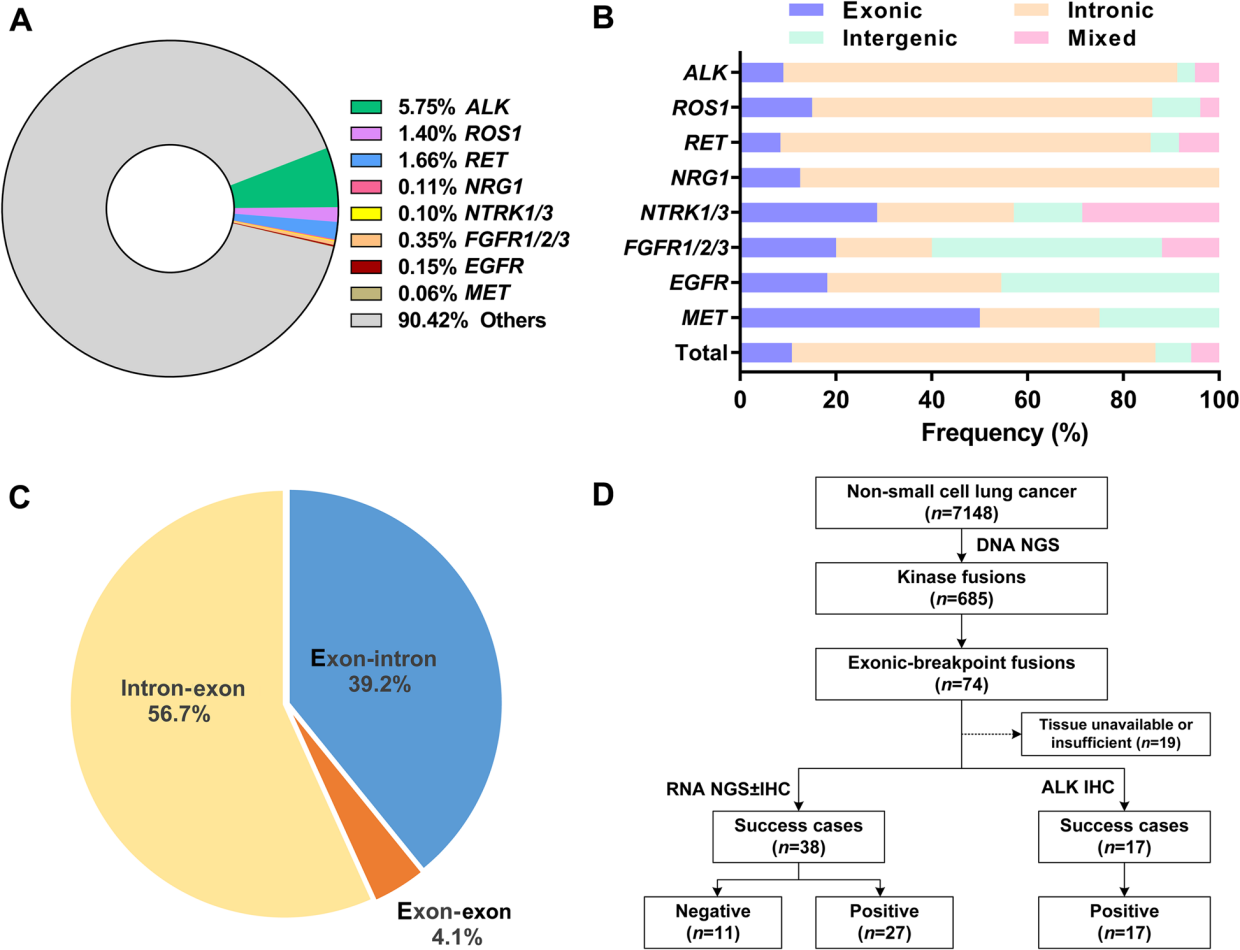
Distribution and characteristics of exonic-breakpoint fusions identified by DNA NGS in NSCLCs. **A** Distribution of kinase fusions identified by DNA NGS in NSCLCs. **B** Distribution of intronic-, intergenic-, exonic-, and mixed-breakpoint fusions in fusion-positive NSCLCs. **C** Classification of exonic-breakpoint fusions. **D** Flowchart showing molecular analyses of exonic-breakpoint fusions in our cohort

Tumor tissues from 7148 NSCLC patients were interrogated using DNA NGS. Most of these patients (6615/7148, 89.7%) were diagnosed with adenocarcinoma. The patient characteristics are listed in Additional file 1: Table S2. Kinase fusions, including *ALK*, *ROS1*, *RET*, *NRG1*, *NTRK1*, *NTRK3*, *FGFR1*, *FGFR2*, *FGFR3*, *EGFR*, and *MET*, were identified in 685 (9.6%) cases. *ALK* was the most commonly detected fusion gene, followed by *RET* and *ROS1* (Fig. 1A). According to the genome base position of the breakpoint, fusions can be divided into four subtypes: intronic-breakpoint (“intron-intron” fusions), intergenic-breakpoint (“intron-intergenic,” “intergenic-intron,” or “intergenic-intergenic” fusions), exonic-breakpoint (“intron-exon,” “exon-intron,” or “exon-exon” fusions), and mixed-breakpoint fusions (“exon-intergenic” fusions, “intergenic-exon” fusions, or multiple fusions with two or more breakpoint subtypes). Overall, intronic-, intergenic-, exonic-, and mixed-breakpoint fusions were identified in 520 (75.9%), 51 (7.4%), 74 (10.8%), and 40 (5.8%) cases, respectively. The ratio of exonic-breakpoint fusion events was higher for uncommon fusions than for *ALK*, *ROS1*, and *RET* fusions (21.8% vs. 9.8%, *P* = 0.006, Fig. 1B), although no marked differences in clinicopathological characteristics were found between exonic-breakpoint fusions and other fusion subtypes (Additional file 1: Table S3). Paired samples from the same patient, including paired primary and metastatic tumors (*n* = 4), paired different metastatic tumors (*n* = 1), paired tissue and plasma samples (*n* = 2), and paired pre- and post-TKI samples (*n*=3), showed the same rearrangement involving exonic breakpoint, further supporting the presence of exonic-breakpoint fusions at the DNA level (Additional file 1: Table S4).

### Classification and validation of exonic-breakpoint fusions

The exonic-breakpoint fusions could be further classified into three categories on the basis of relative genomic locations: (i) “exon-intron” fusions, 39.2% (29/74); (ii) “exon-exon” fusions, 4.1% (3/74); and (iii) “intron-exon” fusions, 56.7% (42/74) (Fig. 1C and Additional file 2: Fig. S1A-C). To verify the function and activity of exonic-breakpoint fusions, RNA NGS and/or IHC were successfully performed in 55 cases with sufficient tissue. Chimeric transcripts/proteins were identified in 44 cases (80%), whereas no expressed fusion transcript/protein was detected in 11 cases (20%, Fig. 1D).

### Possible mechanisms by which exonic-breakpoint fusions generate no aberrant transcript/protein

**Fig. 2:**
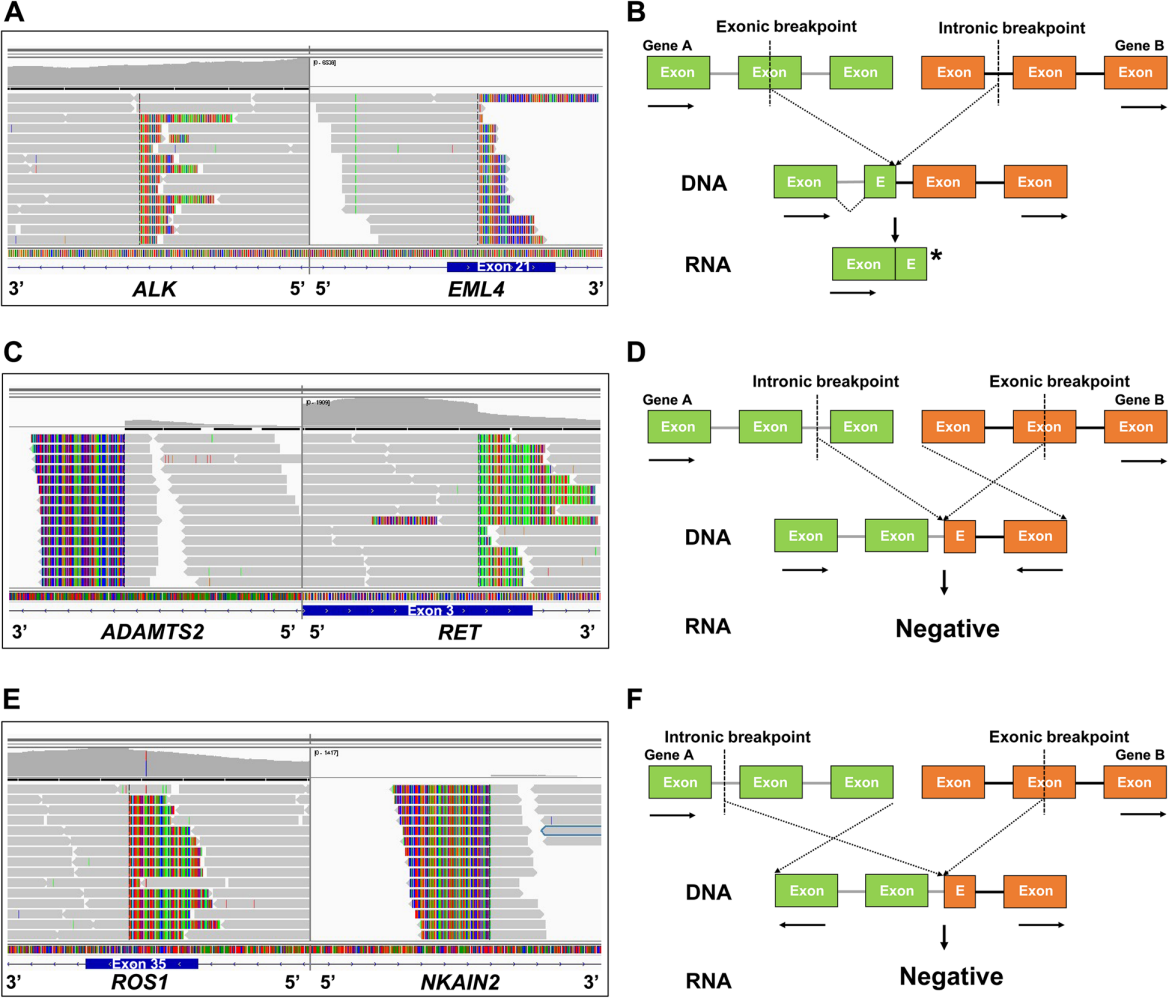
Examples and schematic diagrams of exonic-breakpoint fusions generating no chimeric fusion transcripts. **A** DNA NGS detected an exonic-breakpoint *ALK* fusion involving *EML4* exon 21 and *ALK* intron 19 in P1. **B** Schematic diagram of the predicted fusion detected by DNA NGS but not by RNA NGS due to open reading frame disruption. **C** DNA NGS detected an uncommon exonic-breakpoint *RET* fusion involving *ADAMTS2* intron 10 and *RET* exon 3 in P6. **D** Schematic diagram of the predicted fusion detected by DNA NGS but not by RNA NGS due to different transcriptional orientations (5’-5’ fusion). **E** DNA NGS detected an uncommon exonic-breakpoint *ROS1* fusion involving *NKAIN2* intron 6 and *ROS1* exon 35 in P10. **F** Schematic diagram of the predicted fusion detected by DNA NGS but not by RNA NGS due to different transcriptional orientations (3’-3’ fusion). The gray bars indicate sequencing reads that match the reference genome, and multicolored bars indicate mismatched reads (the corresponding partners). The asterisk indicates a premature stop codon. The rectangles indicate exons (E, exon), and the solid lines indicate introns. The arrows indicate the direction of transcription

Among the 11 cases with genomic-positive but RNA/protein-negative fusions, 4 (P1-P4) harbored “exon-intron” fusions involving *ALK* (*n*=2), *NTRK1* (*n*=1), or *FGFR1* (*n*=1) and 1 (P5) harbored an “exon-exon” *ROS1* fusion (Table 1). Open reading frame disruption in the exonic regions of the 5′ portion may be responsible for these nonproductive rearrangements, as an exon disrupting breakpoint may introduce a premature stop codon that leads to the production of a truncated transcript/protein (Fig. 2A, B). Moreover, the remaining 6 cases (P6-P11) harbored “intron-exon” fusions involving *ALK* (*n*=1), *ROS1* (*n*=1), *RET* (*n*=1), *FGFR2* (*n*=1), *MET* (*n*=1), and *EGFR* (*n*=1). All of these specific fusions had a fusion partner that was rare or was never reported (Table 1). Upon manual review in Integrative Genomics Viewer (IGV) [17], we found that the 5′ portion of the kinase genes and the 5′ portion of the partners merged to form “5’-5’ fusions” in 4 cases (P6–P9; Table 1 and Fig. 2C). Similarly, the 3′ portion of the kinase genes and the 3′ portion of the partners merged to form “3’-3’ fusions” in 2 cases (P10–P11; Table 1 and Fig. 2E). The discrepancy between the DNA NGS and RNA NGS/IHC results was likely a result of the formation of antisense rearrangements in which the kinase genes and partners were transcribed in different orientations, resulting in no aberrant transcript/protein (Fig. 2D, F).

**Table 1:** Inconsistent results observed at the DNA and RNA/protein levels in patients with exonic-breakpoint fusions

Case	Sex	Age	Diagnosis	DNA NGS		RNA NGS	FISH	IHC
				Fusion	Other variants			
**Open reading frame disruption**								
P1	M	50	ADC	*EML4-ALK* (exon 21: intron 19)	*TP53* p.L194P	−	+	−
P2	M	66	ADC	*EML4-ALK* (exon 21: intron 19)	None	−	+	−
P3	M	66	ADC	*LMNA-NTRK1* (exon 8: intron 11)	*FGFR2* p.S799fs	−	N/A	−
P4	M	62	ADC	*FGFR1-NSD3* (exon 14: intron 19)	None	−	N/A	N/A
P5	F	46	ADC	*CD74-ROS1* (exon 7: exon 33)	*CTNNB1* p.S45P	−	+	−
**5’-5’ fusion**								
P6	M	54	ADC	*ADAMTS2-RET* (intron 10: exon 3)	*EML4-ALK*	*EML4-ALK*	N/A	N/A
P7	M	81	ADC	*WNT2-MET* (intron 2: exon 11)	*TP53* p.H179R, *RB1* p.K143fs	−	N/A	N/A
P8	F	58	ADC	*NAMPT-EGFR* (intron 1: exon 17)	None	−	N/A	N/A
P9	M	66	ADC	*FGFR2-FAM170B-AS1* (intron 17: exon 1)	*TP53* p.R337C	−	N/A	N/A
**3’-3’ fusion**								
P10	F	76	ADC	*NKAIN2-ROS1* (intron 6: exon 35)	None	−	N/A	−
P11	F	67	ADC	*ZDHHC17-ALK* (intron 1: exon 2)	*EGFR* p.E709_T710>D	−	N/A	−

*F* Female, *M* Male, *ADC* Adenocarcinoma, *N/A* Not available, *NGS* Next-generation sequencing, *FISH* Fluorescence in situ hybridization, *IHC* Immunohistochemistry, *−* negative, *+* positive

### Possible mechanisms by which exonic-breakpoint fusions produce functional transcripts/proteins

**Fig. 3:**
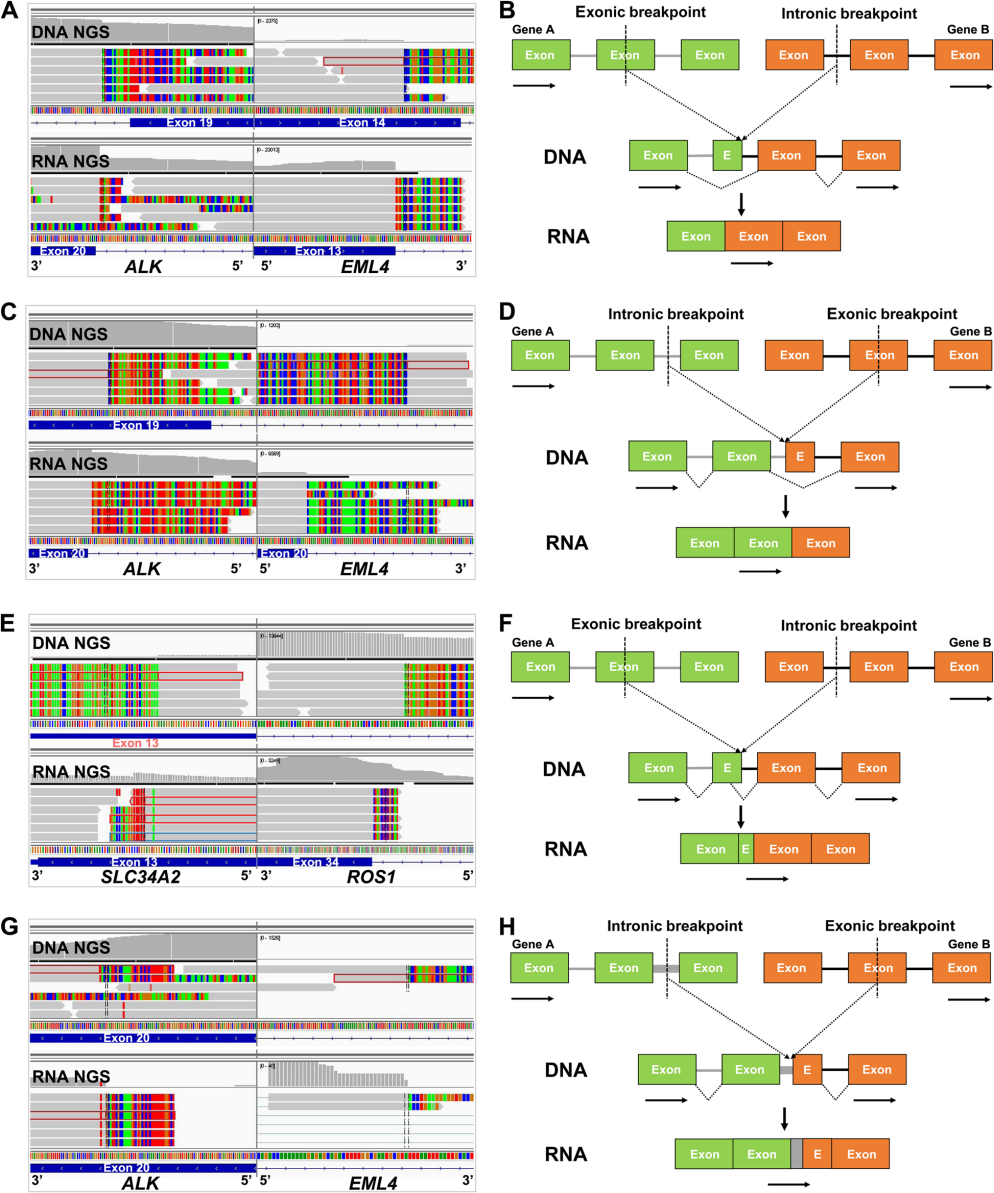
Examples and schematic diagrams of exonic-breakpoint fusions generating functional transcripts. **A** In P12, DNA NGS detected an exonic-breakpoint *ALK* fusion involving *EML4* exon 14 and *ALK* intron 19, whereas RNA NGS revealed an *ALK* fusion involving *EML4* exon 13 and *ALK* exon 20. **B** Schematic diagram showing that the breakpoint at the transcript level differs from that predicted by the genomic breakpoint in the “exon-intron” fusion due to exon skipping. **C** In P23, DNA NGS showed an exonic-breakpoint *ALK* fusion involving *EML4* intron 20 and *ALK* exon 19, whereas RNA NGS detected an *ALK* fusion involving *EML4* exon 20 and *ALK* exon 20. **D** Schematic diagram showing that the breakpoint at the transcript level differs from that predicted by the genomic breakpoint in the “intron-exon” fusion due to exon skipping. **E** In P37, DNA NGS showed an exonic-breakpoint *ROS1* fusion involving *SLC34A2* exon 13 and *ROS1* intron 33, and RNA NGS revealed a *ROS1* fusion involving *SLC34A2* exon 13 and *ROS1* exon 34. **F** Schematic diagram showing that the breakpoint at the transcript level differs from that predicted by the genomic breakpoint due to alternative splice site selection. **G** In P38, DNA NGS detected an exonic-breakpoint *ALK* fusion involving *EML4* intron 6 and *ALK* exon 20, whereas RNA NGS revealed an *ALK* fusion involving *EML4* intron 6 and *ALK* exon 20. **H** Schematic diagram showing that the breakpoint at the transcript level differs from that predicted by the genomic breakpoint due to intron retention. The gray bars indicate sequencing reads that match the reference genome, and multicolored bars indicate mismatched reads (the corresponding partners). The rectangles indicate exons (E, exon), and the solid lines indicate introns. The arrows indicate the direction of transcription

Among the 44 cases with consistent positive results identified by DNA NGS and RNA NGS/IHC, 19 harbored “exon-intron” fusions involving *ALK* (*n*=13), *ROS1* (*n*=3), *RET* (*n*=1), *NRG1* (*n*=1), and *EGFR* (*n*=1) and 25 harbored “intron-exon” fusions involving *ALK* (*n*=16), *ROS1* (*n*=5), and *RET* (*n*=4) (Table 2). A manual review of matched DNA and RNA NGS results in IGV was conducted to reveal the possible mechanisms. For P12, with an “exon-intron” fusion (*EML4* exon 14-*ALK* intron 19), DNA NGS indicated the genomic breakpoint in *EML4* to be located in the middle region of exon 14, but RNA NGS revealed the breakpoint at the transcript level to be located in exon 13 (Fig. 3A), possibly due to the lack of the 3′ acceptor splice site of the *EML4* exon 14 that led to exon skipping (Fig. 3B). Indeed, we found 11 such cases in our cohort (Table 2). Similarly, DNA NGS showed the genomic breakpoint in *ALK* to be located in the middle region of exon 19 in P23, which harbored an “intron-exon” fusion (*EML4* intron 20-*ALK* exon 19). Nevertheless, exon 19 of *ALK* was spliced out of the fusion transcript, as revealed by RNA NGS (Fig. 3C). This phenomenon was observed in 12 cases in our cohort, possibly due to exon skipping (Table 2), as the disrupting exon of the 3′ portion lacked a 5′ donor splice site (Fig. 3D). In particular, primary/reciprocal *ROS1* fusions were identified by DNA NGS in P26; the reciprocal *ROS1* fusion (*ROS1-CLK1*) was also detected by RNA NGS, although its biological impact on cellular function remained to be confirmed (Table 2). Moreover, uncommon *ALK* fusions were detected in both P35 and P36 (P35: *C2orf91* exon 4-*ALK* intron 19; P36: *CLHC1* intron 4-*ALK* exon 19; Table 2) by DNA NGS, but the canonical *EML4-ALK* fusion transcript was identified in these two cases (Additional file 2: Fig. S2A and C). This indicates the involvement of complex rearrangements constituting multiple fusion junctions and rare genes at the DNA level. Nevertheless, these rare genes, even though they showed different transcriptional orientations from the kinase genes (P36), were spliced out during transcription (Additional file 2: Fig. S2B and D).

**Table 2:** Consistent results observed at the DNA and RNA/protein levels in patients with exonic-breakpoint fusions

Case	Sex	Age	Diagnosis	DNA NGS		RNA NGS	FISH	IHC
				Fusion	Other variants			
**Exon skipping (5′ gene)**								
P12	F	63	ADC	*EML4-ALK* (exon 14: intron 19)	*TP53* p.E171K	*EML4-ALK* (exon 13: exon 20)	N/A	+
P13	M	57	ADC	*EML4-ALK* (exon 14: intron 19)	None	*EML4-ALK* (exon 13: exon 20)	N/A	+
P14	F	35	ADC	*EML4-ALK* (exon 21: intron 19)	None	*EML4-ALK* (exon 20: exon 20)	N/A	+
P15	F	69	ADC	*EML4-ALK* (exon 14: intron 19)	None	*EML4-ALK* (exon 13: exon 20)	N/A	+
P16	F	44	ADC	*EML4-ALK* (exon 21: intron 19)	None	*EML4-ALK* (exon 20: exon 20)	N/A	+
P17	F	58	ADC	*EML4-ALK* (exon 21: intron 19)	*PTEN* p.E99*	*EML4-ALK* (exon 20: exon 20)	N/A	+
P18	F	66	ADC	*CD74-ROS1* (exon 7: intron 33)	*TP53* p.R342fs	*CD74-ROS1* (exon 6: exon 34)	N/A	3+
P19	F	72	ADC	*TPM3-ROS1* (3′UTR: intron 34)	None	*TPM3-ROS1* (exon 6: exon 35)	N/A	1+
P20	F	64	ADC	*KIF5B-RET* (exon 17: intron 11)	*TP53* p.I232S	*KIF5B-RET* (exon 16: exon 12)	N/A	3+
P21	F	70	ADC	*CD74-NRG1* (exon 7: intron 5)	None	*CD74-NRG1* (exon 6: exon 6)	N/A	N/A
P22	M	55	ADC	*EGFR-BCAR4* (exon 27: intron 2)	None	*EGFR-BCAR4* (exon 26: exon 3)	N/A	N/A
**Exon skipping (3′ gene)**								
P23	M	63	ADC	*EML4-ALK* (intron 20: exon 19)	None	*EML4-ALK* (exon 20: exon 20)	+	+
P24	M	70	ADC	*EML4-ALK* (intron 6: exon 18)	None	*EML4-ALK* (exon 6: exon 20); *EML4-ALK* (intron 6: exon 20)	N/A	+
P25	F	63	ADC	*EML4-ALK* (intron 6: exon 19)	None	*EML4-ALK* (exon 6: exon 20)	N/A	+
P26	F	72	ADC	*CD74-ROS1* (intron 6: exon 32); *ROS1*-*CLK1* (intron 32: exon 4)	None	*CD74-ROS1* (exon 6: exon 34); *ROS1-CLK1* (exon 32: exon 3)	+	1+
P27	M	56	ADC	*CLTC-ROS1* (intron 31: exon 34)	*TP53* p.G108S	*CLTC-ROS1* (exon 31: exon 35)	N/A	N/A
P28	F	78	ADC	*CD74-ROS1* (intron 6: exon 33)	None	*CD74-ROS1* (exon 6: exon 34)	N/A	3+
P29	M	66	ADC	*EZR-ROS1* (intron 10: exon 32)	*TP53* p.R196*	*EZR-ROS1* (exon 10: exon 34)	N/A	N/A
P30	F	58	ADC	*EZR-ROS1* (intron 10: exon 32)	None	*EZR-ROS1* (exon 9: exon 34)	+	3+
P31	F	40	LCNEC	*KIF5B-RET* (intron 15: exon 11)	*STK11* p.G56_K64del	*KIF5B-RET* (exon 15: exon 12)	N/A	2+
P32	F	32	ADC	*KIF5B-RET* (intron 15: exon 11)	*RB1* p.S842fs, *TP53* p.Y220C	*KIF5B-RET* (exon 15: exon 12)	N/A	3+
P33	M	65	ADC	*KIF5B-RET* (intron 15: exon 11)	*TP53* p.C275G	*KIF5B-RET* (exon 15: exon 12)	N/A	3+
P34	F	46	ADC	*KIF5B-RET* (intron 15: exon 11)	None	*KIF5B-RET* (exon 15: exon 12)	N/A	2+
**Exon skipping in complex rearrangements**								
P35	M	59	ADC	*C2orf91-ALK* (exon 4: intron 19)	None	*EML4-ALK* (exon 20: exon 20)	N/A	N/A
P36	M	57	ADC	*CLHC1-ALK* (intron 4: exon 19)	None	*EML4-ALK* (exon 13: exon 20)	N/A	+
**Alternative 5′splice site**								
P37	F	60	ADC	*SLC34A2-ROS1* (exon 13: intron 33)	None	*SLC34A2-ROS1* (exon 13: exon 34)	+	3+
**Intron retention**								
P38	M	76	ADC	*EML4-ALK* (intron 6: exon 20)	None	*EML4-ALK* (intron 6: exon 20)	N/A	+
**Unknown mechanisms**								
P39	F	50	ADC	*EML4-ALK* (exon 21: intron 19)	None	N/A	N/A	+
P40	M	54	ADC	*EML4-ALK* (exon 14: intron 19)	None	N/A	N/A	+
P41	M	55	ADC	*EML4-ALK* (exon 21: intron 19)	*CDKN2A* p.E26fs, *NF1* p.D618fs	N/A	+	+
P42	F	66	ADC	*EML4-ALK* (exon 14: intron 19)	None	N/A	N/A	+
P43	F	26	ADC	*PCARE-ALK* (exon 1: intron 19)	None	N/A	N/A	+
P44	M	64	ADC	*EML4-ALK* (exon 14: intron 19)	None	N/A	N/A	+
P45	M	70	ADC	*KIF5B-ALK* (intron 20: exon 20)	*TP53* p.E286K	N/A	+	+
P46	F	51	ADC	*PPP1CB-ALK* (intron 4: exon 20)	None	N/A	N/A	+
P47	F	45	ADC	*EML4-ALK* (intron 6: exon 19)	None	N/A	N/A	+
P48	F	65	ADC	*KIF5B-ALK* (intron 15: exon 20)	*TP53* p.K139N	N/A	N/A	+
P49	F	63	ADC	*EML4-ALK* (intron 6: exon19)	None	N/A	N/A	+
P50	M	41	ADC	*FARS2-ALK* (intron 1: exon 24)	None	N/A	N/A	+
P51	M	59	ADC	*PICALM-ALK* (intron 19: exon 20)	None	N/A	N/A	+
P52	M	33	ADC	*EML4-ALK* (intron 4: exon 19)	None	N/A	N/A	+
P53	F	63	ADC	*EML4-ALK* (intron 6: exon 17)	None	N/A	N/A	+
P54	F	65	ADC	*EML4-ALK* (intron 19: exon 20)	None	N/A	N/A	+
P55	M	34	ADC	*EML4-ALK* (intron 14: exon 20)	*TP53* p.H179Y	N/A	N/A	+

*F* Female, *M* Male, *ADC* Adenocarcinoma, *LCNEC* Large cell neuroendocrine carcinoma, *N/A* Not available, *NGS* Next-generation sequencing, *FISH* Fluorescence in situ hybridization, *IHC* Immunohistochemistry, *+* positive, *1+* weak staining, *2+* moderate staining, *3+* strong staining

In addition, a rearrangement of *SLC34A2* exon 13-*ROS1* intron 33 was detected at the DNA level in P37, and RNA NGS revealed that the breakpoint at the transcript level was in *SLC34A2* exon 13, upstream of the predicted breakpoint detected by DNA NGS (Fig. 3E and Table 2). Alternative donor site selection involving a cryptic splice site located within exon 13 of *SLC34A2* might have contributed to the formation of an in-frame fusion transcript (Fig. 3F). In P38, the *EML4* intron 6-*ALK* exon 20 rearrangement detected at the DNA level also produced an *EML4* intron 6-*ALK* exon 20 fusion transcript (Fig. 3G and Table 2). The same breakpoint location was observed between matched DNA NGS and RNA NGS results, probably due to intron retention that the intron sequence was integrated into the transcript (Fig. 3H).

### Clinical outcomes of targeted therapy in patients with exonic-breakpoint fusions

**Fig. 4:**
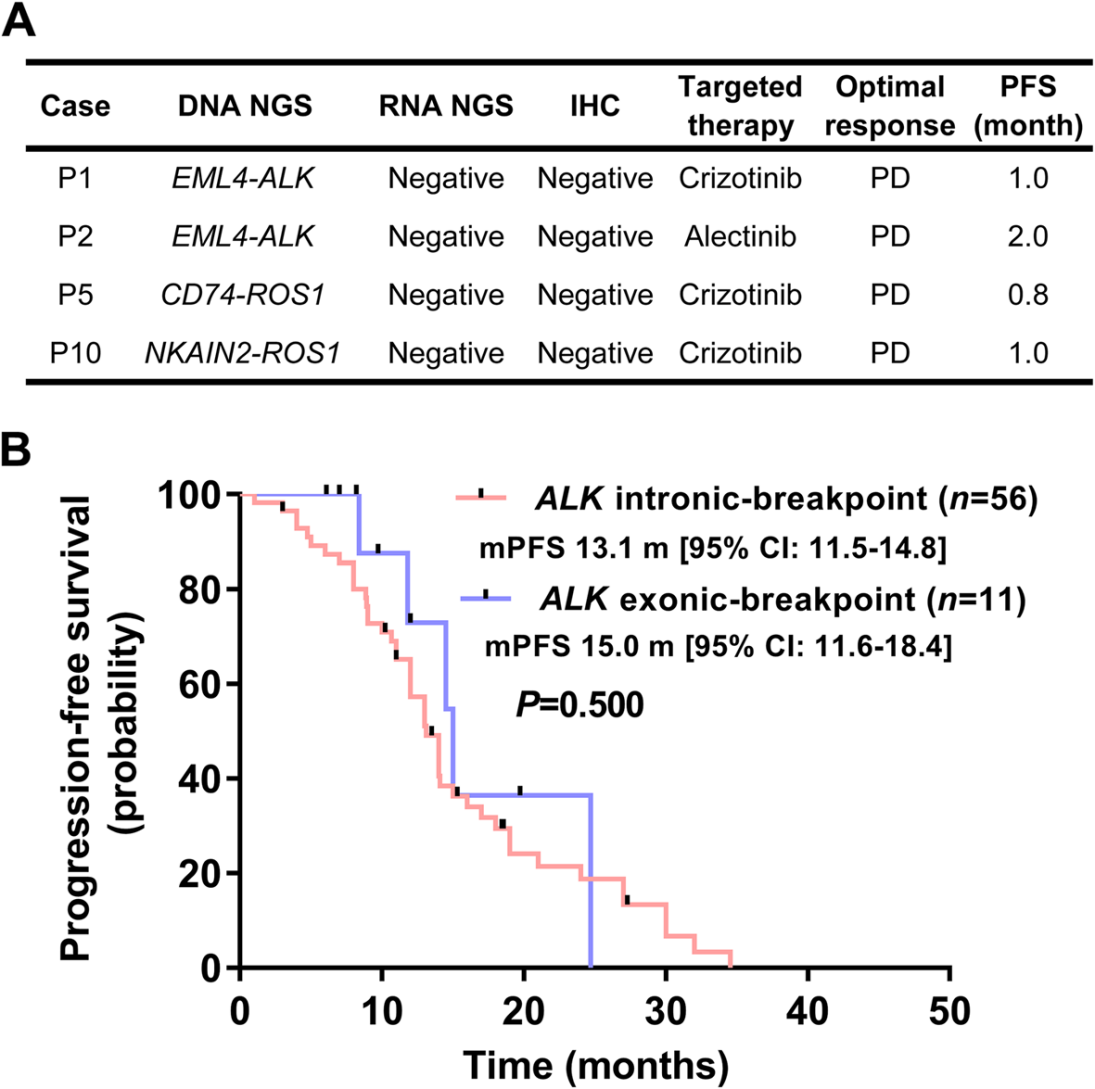
Evaluation of clinical outcomes of patients who received matched targeted therapies. **A** Evaluation of the clinical outcomes of patients with exonic-breakpoint fusions detected by DNA NGS but not by RNA NGS and IHC. **B** Survival curves for exonic-breakpoint and intronic-breakpoint *ALK* fusions among patients with RNA NGS/IHC-validated ALK fusions

Four patients with genomic-positive but RNA/protein-negative *ALK* or *ROS1* fusions received targeted agents (crizotinib or alectinib) as first-line treatment, but showed PD within 2 months because no expressed fusion transcript/protein was produced (Fig. 4A). Among those with genomic- and RNA/protein-positive fusions, 19 received a matched targeted therapy, including agents against *ALK* (*n*=15), *ROS1* (*n*=2), and *RET* (*n*=2), and almost all of them benefited from this treatment (Additional file 1: Table S5). Among them, 11 patients harboring exonic-breakpoint *ALK* fusions who received crizotinib as a first-line treatment were further analyzed, together with 56 harboring intronic-breakpoint *ALK* fusions. All these cases were confirmed to be ALK positive by RNA NGS or IHC, and there were no marked differences in the baseline features of the two groups (Additional file 1: Table S6). Compared with patients harboring intronic-breakpoint *ALK* fusions, those harboring exonic-breakpoint *ALK* fusions exhibited no difference in the ORR (76.8%, 95% CI: 64.2–85.9% vs. 81.8%, 95% CI: 52.3–94.9%, *P* = 0.714; Additional file 1: Table S7). Moreover, no difference in median PFS was observed between patients with intronic-breakpoint *ALK* fusions (13.1 months, 95% CI: 11.5–14.8) and those with exonic-breakpoint *ALK* fusions (15.0 months, 95% CI: 11.6–18.4, *P*=0.500, Fig. 4B). Additionally, although no validation assays were available for the 19 patients harboring exonic-breakpoint fusions, 4 with *ALK* or *ROS1* fusions treated with crizotinib or alectinib showed a durable response (10.1–23.8 months, Additional file 1: Table S5), strongly suggesting the presence of active fusion events in these 4 cases. However, two patients with exonic-breakpoint *ROS1* fusions failed to respond to crizotinib therapy, indicating that the fusion events in these 2 cases may have been silenced with no detectable transcript or clinical relevance.

## Discussion

NGS has greatly improved the molecular diagnosis of cancer patients harboring diverse genetic aberrations [18]. With DNA mutations such as those in *EGFR* and Kirsten rat sarcoma viral oncogene homolog (*KRAS*) constituting a major percentage of Chinese NSCLC cases, DNA NGS is the first choice for patients who have never undergone genetic testing. However, in previous studies, we found that the capability of DNA NGS in identifying and characterizing fusion events is limited, especially when uncommon fusions are concerned [9, 10]. In the present study, through a systematic comparison of DNA NGS results with RNA NGS/IHC results, as well as analyses of matched targeted therapy efficacy, we found fusions involving exonic breakpoints to be difficult to interpret only from DNA sequencing data, as functional fusion transcripts/proteins may or may not be produced, thus confounding the oncologists in decision-making for treatment prioritization.

The genomic breakpoints of fusions usually occur in intronic regions, possibly because for most genes, introns are much longer than exons [19]. Despite this bias for intronic breakpoints, exonic breakpoints have been reported in some fusions, including *ALK*, *ROS1*, and *FGFRs* [20–23]. However, the characteristics and functions of exonic-breakpoint fusions in NSCLC remain largely unknown. In our study, intronic-breakpoint fusions constituted a large portion (75.9%) of our cohort of fusion-positive NSCLC patients, and exonic-breakpoint fusions comprised only 7.4%. Intronic-breakpoint fusions are usually transcribed into in-frame fusion transcripts because the coding sequences are rarely disrupted [9]. However, up to 20% (11/55) of exonic-breakpoint fusions in our study were nonproductive. Exonic breakpoints can occur in the 5′ or 3′ portion of a gene or in both, leading to the formation of “exon-intron,” “intron-exon,” or “exon-exon” fusions. Notably, breakpoints that occur in the exon of the 5′ portion of a gene have a high chance (two-in-three chance) of disrupting the coding sequence, thus introducing a premature stop codon and generating a nontranslated transcript. However, for fusions that disrupt an exon in the 3′ portion of a gene, nonproductive rearrangements are formed mainly because the two merged genes are in different transcriptional orientations. These antisense rearrangements can also be detected in intronic-breakpoint fusion events, and in this and other studies, all these antisense rearrangements have a rare partner [24]. Thus, a novel rearrangement identified by DNA sequencing potentially requires knowledge of the transcriptional orientation of the respective gene, and it is best to perform a confirmatory RNA or protein assay to validate the results.

The fate of an exonic-breakpoint fusion to be transcribed (or not) into aberrant transcripts may be regulated by multiple mechanisms. Alternative splicing may constitute an important biological process that contributes to the production of in-frame fusion transcripts from exonic-breakpoint fusions; indeed, in our study, the genomic breakpoint position did not logically predict the breakpoint position at the transcript level in all RNA-confirmed cases. Alternative splicing is a critical posttranscriptional regulatory mechanism in many types of cancer, and five major forms of alternative splicing have been defined: exon skipping, intron retention, mutually exclusive exons, alternative 5′ splice sites, and alternative 3′ splice sites [25, 26]. Exon skipping is the most common alternative splicing event in humans, followed by alternative 5′ donor and 3′ acceptor site selection and intron retention [27]. Through comparison of matched DNA and RNA NGS results, we found that 92.6% (25/27) of exonic-breakpoint fusions generated in-frame fusion transcripts due to exon skipping. In particular, genomic fusion partners identified by DNA NGS were uncommon genes, but the fusion partner at the RNA level was *EML4* in P35 and P36. One likely cause of such an event is chromothripsis involving many small fragments colocalized to a confined genomic region [28]. However, regions of uncommon genes are removed during transcription. In P37, one exonic-breakpoint *ROS1* fusion generated an in-frame fusion transcript, possibly because of alternative donor site selection. This splicing process has also been reported in a recent study, in which concurrent *EML4-ALK* v3a and v3b variants are detected in *EML4-ALK* v3 NSCLC [29]. Moreover, the same position was observed between the genomic breakpoint and breakpoint at the transcript level in P38, which led to a novel *EML4-ALK* variant, possibly owing to intron retention in which the disrupting intron of *EML4* and disrupting exon of *ALK* were both retained in the chimeric fusion transcript.

Fusion genes drive cancer cell growth and survival mainly through the formation of deleterious chimeric proteins. Several TKIs have been developed to target the kinase domain of these chimeric proteins [30]. Theoretically, nonproductive rearrangements involving exonic breakpoints do not respond well to a matched targeted therapy, but clinical validation is needed. In this study, patients with genomic-positive but RNA/protein-negative fusions who received a relevant targeted therapy showed poor clinical outcomes. However, when genomic- and RNA/protein-positive cases were analyzed, a high ORR and long-term median PFS were observed. In particular, similar ORRs and median PFS were found between patients with exonic-breakpoint *ALK* fusions and those with intronic-breakpoint *ALK* fusions when ALK RNA- or protein-validated patients who received first-line crizotinib were analyzed. These data further confirm the potential inaccuracy and unreliability of exonic-breakpoint fusions in predicting the relevant biological outcomes of targeted therapy. Notably, in contrast to constitutive splicing that removes introns and ligates consecutive exons to form expressed fusion transcripts from intronic-breakpoint fusions, alternative splicing may be responsible for generating functional fusion transcripts from exonic-breakpoint fusions. Thus, agents targeting alternative splicing combined with matched TKIs may add value for NSCLC patients harboring RNA/protein-confirmed exonic-breakpoint fusions, which may be a promising research direction for further studies [31].

Our study had some limitations. First, although simultaneous DNA and RNA NGS may accurately illustrate the results of exonic-breakpoint fusions and reveal underlying mechanisms, the instability of RNA is the main drawback for RNA NGS [32]. Second, the clinical relevance of exonic-breakpoint fusions with targeted therapy was explored mainly on the basis of crizotinib in a relatively small number of cases. Future studies involving newer generations of TKIs and a larger population are needed to validate the conclusion.

## Conclusions

Our study demonstrates that exonic-breakpoint fusions identified by DNA NGS might be unable to produce fusion transcripts/proteins due to open reading frame disruption or different fusion gene transcriptional orientations, or might produce functional fusion transcripts/proteins owing to alternative splicing. Therefore, when fusions involving exonic breakpoints detected by DNA sequencing occur in the clinic, combined detection of RNA or protein should be implemented to confirm the existence of in-frame functional fusions.

## Supplementary Information

**Additional file 1: Table S1.** The gene panel used in RNA NGS. **Table S2.** Characteristics of NSCLC patients who underwent DNA NGS. **Table S3.** Clinicopathological features of NSCLC cases with exonic- or intronic/intergenic/mixed-breakpoint fusions. **Table S4.** Exonic-breakpoint fusions identified by DNA NGS in paired samples from the same patient. **Table S5.** The clinical outcomes of matched targeted therapy in NSCLC cases with exonic-breakpoint fusions. **Table S6.** Baseline characteristics of 67 NSCLC patients with RNA NGS/IHC-confirmed ALK fusions who received first-line crizotinib. **Table S7.** Clinical response to crizotinib treatment for NSCLC patients with intronic-breakpoint or exonic-breakpoint *ALK* fusions confirmed by RNA NGS/IHC.

**Additional file 2: Figure S1.** Schematic diagrams showing the formation of (A) “exon-intron”, (B) “exon-exon” and (C) “intron-exon” fusions at the DNA level. The rectangles indicate exons (E, exon), and the solid lines indicate introns. The arrows indicate the direction of transcription. **Figure S2.** Examples and schematic diagrams of exonic-breakpoint fusions that generate functional transcripts with different partners. (A) In P35, DNA NGS showed an exonic-breakpoint *ALK* fusion involving *C2orf91* exon 4 and *ALK* intron 19, whereas RNA NGS detected an *ALK* fusion involving *EML4* exon 20 and *ALK* exon 20. (B) Schematic diagram of a possible mechanism leading to *C2orf91*-*ALK* detected by DNA NGS, but *EML4*-*ALK* detected by RNA NGS. (C) In P36, DNA NGS detected an exonic-breakpoint *ALK* fusion involving *CLHC1* intron 4 and *ALK* exon 19, whereas RNA NGS revealed an *ALK* fusion involving *EML4* exon 13 and *ALK* exon 20. (D) Schematic diagram of a possible mechanism leading to *CLHC1-ALK* detected by DNA NGS, but *EML4*-*ALK* detected by RNA NGS. The gray bars indicate sequencing reads that match the reference genome, and multicolored bars indicate mismatched reads (the corresponding partners). The rectangles indicate exons (E, exon), and the solid lines indicate introns. The arrows indicate the direction of transcription.

## Data Availability

The datasets used and/or analyzed during the current study are available from the corresponding author on reasonable request.

## Abbreviations

*ALK*: Anaplastic lymphoma kinase
CR: Complete response
*EGFR*: Epidermal growth factor receptor
*FGFR*: Fibroblast growth factor receptor
FISH: Fluorescence in situ hybridization
HE: Hematoxylin and eosin
IGV: Integrative Genomics Viewer
IHC: Immunohistochemistry
*KRAS*: Kirsten rat sarcoma viral oncogene homolog
*MET*: Mesenchymal-epithelial transition factor
NGS: Next-generation sequencing
*NRG1*: Neuregulin 1
NSCLC: Non-small cell lung cancer
*NTRK*: Neurotrophic receptor tyrosine kinase
ORR: Objective response rate
PD: Progressive disease
PFS: Progression-free survival
PR: Partial response
*RET*: Rearranged during transfection
*ROS1*: V-ros UR2 sarcoma virus oncogene homolog 1
SD: Stable disease
TKI: Tyrosine kinase inhibitor
VAF: Variant allele frequency
