# Supplementary material for: Reliability analysis of exonic-breakpoint fusions identified by DNA sequencing for predicting the efficacy of targeted therapy in non-small cell lung cancer
Source: BMC Med. 2022 May 10;20:160. doi: 10.1186/s12916-022-02362-9 (PMC9087946; doi:10.1186/s12916-022-02362-9)
Supplement: Supplementary file 1 — Additional file 1: Table S1. The gene panel used in RNA NGS. Table S2. Characteristics of NSCLC patients who underwent DNA NGS. Table S3. Clinicopathological features of NSCLC cases with exonic- or intronic/intergenic/mixed-breakpoint fusions. Table S4. Exonic-breakpoint fusions identified by DNA NGS in paired samples from the same patient. Table S5. The clinical outcomes of matched targeted therapy in NSCLC cases with exonic-breakpoint fusions. Table S6. Baseline characteristics of 67 NSCLC patients with RNA NGS/IHC-confirmed ALK fusions who received first-line crizotinib. Table S7. Clinical response to crizotinib treatment for NSCLC patients with intronic-breakpoint or exonic-breakpoint ALK fusions confirmed by RNA NGS/IHC. [file 12916_2022_2362_MOESM1_ESM.docx]

**Table S1.** The gene panel used in RNA NGS.

| *ABL1* | *AKT1* | *AKT3* | *ALK* | *ARHGAP26* | *AXL* | *BRAF* | *BRD3* |
| --- | --- | --- | --- | --- | --- | --- | --- |
| *BRD4* | *CALCA* | *CAMTA1* | *CCNB3* | *CCND1* | *CIC* | *CTNNB1* | *DDR2* |
| *EGFR* | *EPC1* | *ERBB2* | *ERBB4* | *ERG* | *ESR1* | *ESRRA* | *ETV1* |
| *ETV4* | *ETV5* | *ETV6* | *EWSR1* | *FGFR1* | *FGFR2* | *FGFR3* | *FGR* |
| *FOXO1* | *FUS* | *GLI1* | *GNAS* | *HMGA2* | *HRAS* | *IDH1* | *IDH2* |
| *INSR* | *JAK2* | *JAZF1* | *KRAS* | *KRT20* | *MAML2* | *MAP2K1* | *MAST1* |
| *MAST2* | *MEAF6* | *MET* | *MKL2* | *MSMB* | *MUSK* | *MYB* | *NCOA2* |
| *NOTCH1* | *NOTCH2* | *NRAS* | *NRG1* | *NTRK1* | *NTRK2* | *NTRK3* | *NUMBL* |
| *PDGFB* | *PDGFRA* | *PDGFRB* | *PIK3CA* | *PKN1* | *PLAG1* | *PPARG* | *PRKCA* |
| *PRKCB* | *PTH* | *PTK2B* | *RAF1* | *RARA* | *RELA* | *RET* | *ROS1* |
| *RSPO2* | *RSPO3* | *SLC5A5* | *SS18* | *STAT6* | *TAF15* | *TCF12* | *TERT* |
| *TFE3* | *TFEB* | *TFG* | *THADA* | *TMPRSS2* | *TTF1* | *YWHAE* |  |

**Table S2.** Characteristics of NSCLC patients who underwent DNA NGS.

| **Patient** | **No (%)** |
| --- | --- |
| **Total** | 7148 |
| **Gender** |  |
| Male | 3891 (54.4) |
| Female | 3257 (45.6) |
| **Age** |  |
| ≥60 | 4013 (56.1) |
| <60 | 3135 (43.9) |
| **Histology** |  |
| Adenocarcinoma | 6615 (89.7) |
| Squamous | 241 (3.4) |
| Other* | 292 (4.1) |
| **TKI therapy** |  |
| Naive | 6785 (94.9) |
| Relapsed | 363 (5.1) |
| NGS, next-generation sequencing; NSCLC, non-small cell lung cancer; TKI, tyrosine kinase inhibitor.  *Other carcinomas included adenosquamous carcinoma (*n*=72), large cell neuroendocrine carcinoma (*n*=85), sarcomatoid carcinoma (*n*=46) and non-small cell lung cancer-not otherwise specified (*n*=89). | |

**Table S3.** Clinicopathological features of NSCLC cases with exonic- or intronic/intergenic/mixed-breakpoint fusions.

| **Characteristics** | **Total (*n*, %)** | **Intronic/intergenic/mixed-breakpoint fusion (*n*, %)** | **Exonic-breakpoint fusion (*n*, %)** | ***P*** |
| --- | --- | --- | --- | --- |
| **Gender** |  |  |  |  |
| Male | 289 (42.2) | 253 (41.4) | 36 (48.6) | 0.234 |
| Female | 396 (57.8) | 358 (58.6) | 38 (51.4) |  |
| **Age** |  |  |  |  |
| ≥60 | 252 (36.8) | 219 (35.8) | 33 (44.6) | 0.140 |
| <60 | 433 (63.2) | 392 (64.2) | 41 (55.4) |  |
| **Histology** |  |  |  |  |
| Adenocarcinoma | 660 (96.4) | 591 (96.7) | 69 (93.2) | 0.649 |
| Squamous | 10 (1.5) | 8 (1.3) | 2 (2.7) |  |
| Other* | 15 (2.2) | 12 (2.0) | 3 (4.1) |  |
| **Smoking history** |  |  |  |  |
| Yes | 220 (32.1) | 198 (32.4) | 22 (29.7) | 0.659 |
| No | 422 (61.6) | 375 (61.4) | 47 (63.5) |  |
| Unknown | 43 (6.3) | 38 (6.2) | 5 (6.8) |  |
| **Stage** |  |  |  |  |
| I-IIIA | 293 (42.8) | 263 (43.0) | 30 (40.5) | 0.681 |
| IIIB/IV | 392 (57.2) | 348 (57.0) | 44 (59.5) |  |
| *Other carcinomas included adenosquamous carcinoma (*n*=2), large cell neuroendocrine carcinoma (*n*=5), sarcomatoid carcinoma (*n*=5) and non-small cell lung cancer-not otherwise specified (*n*=3). | | | | |

**Table S4.** Exonic-breakpoint fusions identified by DNA NGS in paired samples from the same patient.

| **Case** | **Sex** | **Sample type** | **Tumor location** | **DNA NGS** | **VAF** |
| --- | --- | --- | --- | --- | --- |
| **Paired primary and metastatic tumors** | | | | |  |
| P5 | Female | Tissue | Lung | *CD74-ROS1* (exon 7: exon 33) | 32.3% |
|  |  | Tissue | Lymph node | *CD74-ROS1* (exon 7: exon 33) | 14.5% |
| P20 | Female | Tissue | Lung | *KIF5B-RET* (exon 17: intron 11) | 10.8% |
|  |  | Cytological | Pleural effusion | *KIF5B-RET* (exon 17: intron 11) | 23.9% |
| P32 | Female | Tissue | Lung | *KIF5B-RET* (intron 15: exon 11) | 22.1% |
|  |  | Tissue | Lung | *KIF5B-RET* (intron 15: exon 11) | 13.4% |
| P33 | Male | Tissue | Lung | *KIF5B-RET* (intron 15: exon 11) | 10.3% |
|  |  | Tissue | Lymph node | *KIF5B-RET* (intron 15: exon 11) | 9.9% |
| **Paired different metastatic tumors** | | | | |  |
| P12 | Female | Tissue | Lymph node | *EML4-ALK* (exon 14: intron 19) | 43.6% |
|  |  | Cytological | Pleural effusion | *EML4-ALK* (exon 14: intron 19) | 21.6% |
| **Paired tissue and plasma samples** | | | | |  |
| P69 | Male | Tissue | Lung | *CD74-ROS1* (intron 6: exon 33) | 42.8% |
|  |  | Plasma | - | *CD74-ROS1* (intron 6: exon 33) | 2.4% |
| P71 | Male | Cytological | Pleural effusion | *KIF5B-RET* (intron 15: exon 11) | 12.3% |
|  |  | Plasma | - | *KIF5B-RET* (intron 15: exon 11) | 0.2% |
| **Paired pre- and post-TKI samples** | | | | |  |
| P38 | Male | Tissue (pre-TKI) | Lung | *EML4-ALK* (intron 6: exon 20) | 12.2% |
|  |  | Tissue (post-TKI) | Liver | *EML4-ALK* (intron 6: exon 20) | 2.1% |
| P58 | Male | Tissue (pre-TKI) | Lung | *GOPC-ROS1* (exon 4: intron 34) | 93.6% |
|  |  | Tissue (post-TKI) | Brain | *GOPC-ROS1* (exon 4: intron 34) | 21.3% |
| P73 | Female | Tissue (pre-TKI) | Lung | *SLC34A2-ROS1* (3’UTR: intron 31) | 28.6% |
|  |  | Cytological (post-TKI) | Pleural effusion | *SLC34A2-ROS1* (3’UTR: intron 31) | 5.0% |

NGS, next-generation sequencing; VAF, variant allele frequency; TKI, tyrosine kinase inhibitor.

**Table S5.** The clinical outcomes of matched targeted therapy in NSCLC cases with exonic-breakpoint fusions.

| **Case** | **DNA NGS** | **RNA NGS** | **IHC** | **Matched therapy** | **Optimal response** | **PFS (month)** |
| --- | --- | --- | --- | --- | --- | --- |
| **Exonic-breakpoint fusions with RNA/protein confirmation** | | | | | | |
| P12 | *EML4-ALK* (exon 14: intron 19) | *EML4-ALK* (exon 13: exon 20) | + | Alectinib | PR | 16.7 |
| P13 | *EML4-ALK*  (exon 14: intron 19) | *EML4-ALK* (exon 13: exon 20) | + | Crizotinib | SD | 8.4 |
| P20 | *KIF5B-RET* (exon 17: intron 11) | *KIF5B-RET* (exon 16: exon 12) | 3+ | Pralsetinib | PR | NR |
| P23 | *EML4-ALK* (intron 20: exon 19) | *EML4-ALK* (exon 20: exon 20) | + | Crizotinib | PR | NR |
| P25 | *EML4-ALK* (intron 6: exon 19) | *EML4-ALK* (exon 6: exon 20) | + | Alectinib | PR | NR |
| P27 | *CLTC-ROS1*  (intron 31: exon 34) | *CLTC-ROS1* (exon 31: exon 35) | N/A | Crizotinib | PR | 10.9 |
| P28 | *CD74-ROS1*  (intron 6: exon 33) | *CD74-ROS1* (exon 6: exon 34) | 3+ | Crizotinib | PR | 10.1 |
| P31 | *KIF5B-RET* (intron 15: exon 11) | *KIF5B-RET*  (exon 15: exon 12) | 2+ | Selpercatinib | PR | 11.7 |
| P36 | *CLHC1-ALK* (intron 4: exon 19) | *EML4-ALK* (exon 13: exon 20) | + | Alectinib | PR | NR |
| P38 | *EML4-ALK* (intron 6: exon 20) | *EML4-ALK* (intron 6: exon 20) | + | Crizotinib | PR | NR |
| P39 | *EML4-ALK* (exon 21: intron 19) | N/A | + | Crizotinib | PR | 11.8 |
| P40 | *EML4-ALK*  (exon 14: intron 19) | N/A | + | Alectinib | PR | 13.2 |
| P42 | *EML4-ALK*  (exon 21: intron 19) | N/A | + | Crizotinib | PR | NR |
| P43 | *PCARE-ALK*  (exon 1: intron 19) | N/A | + | Crizotinib | PR | NR |
| P45 | *KIF5B-ALK*  (intron 20: exon 20) | N/A | + | Crizotinib | PR | 24.7 |
| P46 | *PPP1CB-ALK* (intron 4: exon 20) | N/A | + | Crizotinib | PR | NR |
| P48 | *KIF5B-ALK*  (intron 15: exon 20) | N/A | + | Crizotinib | PR | NR |
| P52 | *EML4-ALK* (intron 4: exon 19) | N/A | + | Crizotinib | PR | 15.0 |
| P55 | *EML4-ALK*  (intron 14: exon 20) | N/A | + | Crizotinib | SD | 14.5 |
| **Exonic-breakpoint fusions without RNA/protein confirmation** | | | | | | |
| P56 | *EML4-ALK* (exon 15: exon 20) | N/A | N/A | Alectinib | PR | 15.5 |
| P58 | *GOPC-ROS1*  (exon 4: intron 34) | N/A | N/A | Crizotinib | PD | 2.8 |
| P59 | *LRIG3-ROS1* (exon 16: intron 34) | N/A | N/A | Crizotinib | PR | 11.0 |
| P67 | *RDX-ROS1* (intron 10: exon 33) | N/A | N/A | Crizotinib | SD | 23.8 |
| P69 | *CD74-ROS1*  (intron 6: exon 33) | N/A | N/A | Crizotinib | PR | 10.1 |
| P73 | *SLC34A2-ROS1* (3’UTR: intron 31) | N/A | N/A | Crizotinib | PD | 2.0 |

N/A, not available; NGS, next-generation sequencing; IHC, immunohistochemistry; +, positive; 2+, moderate staining; 3+, strong staining; PFS, progression-free survival; NR, not reached; PR, partial response; SD, stable disease; PD, progressive disease.

| **Table S6.** Baseline characteristics of 67 NSCLC patients with RNA NGS/IHC-confirmed ALK fusions who received first-line crizotinib. | | | | |
| --- | --- | --- | --- | --- |
| **Characteristics** | **Total (*n*=67)** | **Intronic-breakpoint *ALK* fusions (*n*=56)*** | **Exonic-breakpoint *ALK* fusions (*n*=11)*** | ***P*** |
| **Age, year (n, %)** | | | |  |
| ≥60 | 18 (26.9) | 14 (25.0) | 4 (36.4) | 0.685 |
| <60 | 49 (73.1) | 42 (75.0) | 7 (63.6) |  |
| **Sex (n, %)** | | | |  |
| Male | 38 (56.7) | 31 (55.4) | 7 (63.6) | 0.862 |
| Female | 29 (43.3) | 25 (44.6) | 4 (36.4) |  |
| **Smoking history (n, %)** | | | |  |
| Yes | 20 (29.9) | 18 (32.1) | 2 (18.2) | 0.572 |
| No | 47 (70.1) | 38 (67.9) | 9 (81.8) |  |
| **Histological types (n, %)** | | | |  |
| Adenocarcinoma | 65 (97.0) | 54 (96.4) | 11 (100) | 1.000 |
| Non-adenocarcinoma | 2 (3.0) | 2 (3.6) | 0 |  |
| **Stage (n, %)** | | | |  |
| IIIB | 12 (17.9) | 10 (17.9) | 2 (18.2) | 0.686 |
| IV | 55 (82.1) | 46 (82.1) | 9 (81.8) |  |
| **ECOG score (n, %)** | | | |  |
| 0-1 | 62 (92.5) | 52 (92.9) | 10 (90.9) | 0.687 |
| 2 | 5 (7.5) | 4 (7.1) | 1 (9.1) |  |
| ***TP53* status (n, %)** | | | | |
| Mutated | 21 (31.3) | 18 (32.1) | 3 (27.3) | 0.970 |
| Wild-type | 46 (68.7) | 38 (67.9) | 8 (72.7) |  |
| *These cases were all ALK positive validated by RNA NGS or/and IHC.  ECOG, Eastern Cooperative Oncology Group. | | | | |

**Table S7.** Clinical response to crizotinib treatment for NSCLC patients with intronic-breakpoint or exonic-breakpoint *ALK* fusions confirmed by RNA NGS/IHC.

| **Response** | **Crizotinib** | | |  |
| --- | --- | --- | --- | --- |
|  | **All (*n*=67)** | **Intronic-breakpoint *ALK* fusions (*n*=56)*** | **Exonic-breakpoint *ALK* fusions (*n*=11)*** | ***P*** |
| **CR (%)** | 0 | 0 | 0 |  |
| **PR (%)** | 52 | 43 | 9 |  |
| **SD (%)** | 13 | 11 | 2 |  |
| **PD (%)** | 2 | 2 | 0 |  |
| **ORR [95% CI]** | 77.6 (66.3-85.9) | 76.8 (64.2-85.9) | 81.8 (52.3-94.9) | 0.714 |

*These cases were all ALK positive validated by RNA NGS or/and IHC.

CR, complete response; PR, partial response; SD, stable disease; PD, progressive disease; ORR, objective response rate; CI, confidence interval.
