# Supplementary material for: Reliability analysis of exonic-breakpoint fusions identified by DNA sequencing for predicting the efficacy of targeted therapy in non-small cell lung cancer
Source: BMC Med. 2022 May 10;20:160. doi: 10.1186/s12916-022-02362-9 (PMC9087946; doi:10.1186/s12916-022-02362-9)
Supplement: Supplementary file 2 — Additional file 2: Figure S1. Schematic diagrams showing the formation of (A) “exon-intron”, (B) “exon-exon” and (C) “intron-exon” fusions at the DNA level. The rectangles indicate exons (E, exon), and the solid lines indicate introns. The arrows indicate the direction of transcription. Figure S2. Examples and schematic diagrams of exonic-breakpoint fusions that generate functional transcripts with different partners. (A) In P35, DNA NGS showed an exonic-breakpoint ALK fusion involving C2orf91 exon 4 and ALK intron 19, whereas RNA NGS detected an ALK fusion involving EML4 exon 20 and ALK exon 20. (B) Schematic diagram of a possible mechanism leading to C2orf91-ALK detected by DNA NGS, but EML4-ALK detected by RNA NGS. (C) In P36, DNA NGS detected an exonic-breakpoint ALK fusion involving CLHC1 intron 4 and ALK exon 19, whereas RNA NGS revealed an ALK fusion involving EML4 exon 13 and ALK exon 20. (D) Schematic diagram of a possible mechanism leading to CLHC1-ALK detected by DNA NGS, but EML4-ALK detected by RNA NGS. The gray bars indicate sequencing reads that match the reference genome, and multicolored bars indicate mismatched reads (the corresponding partners). The rectangles indicate exons (E, exon), and the solid lines indicate introns. The arrows indicate the direction of transcription. [file 12916_2022_2362_MOESM2_ESM.docx]

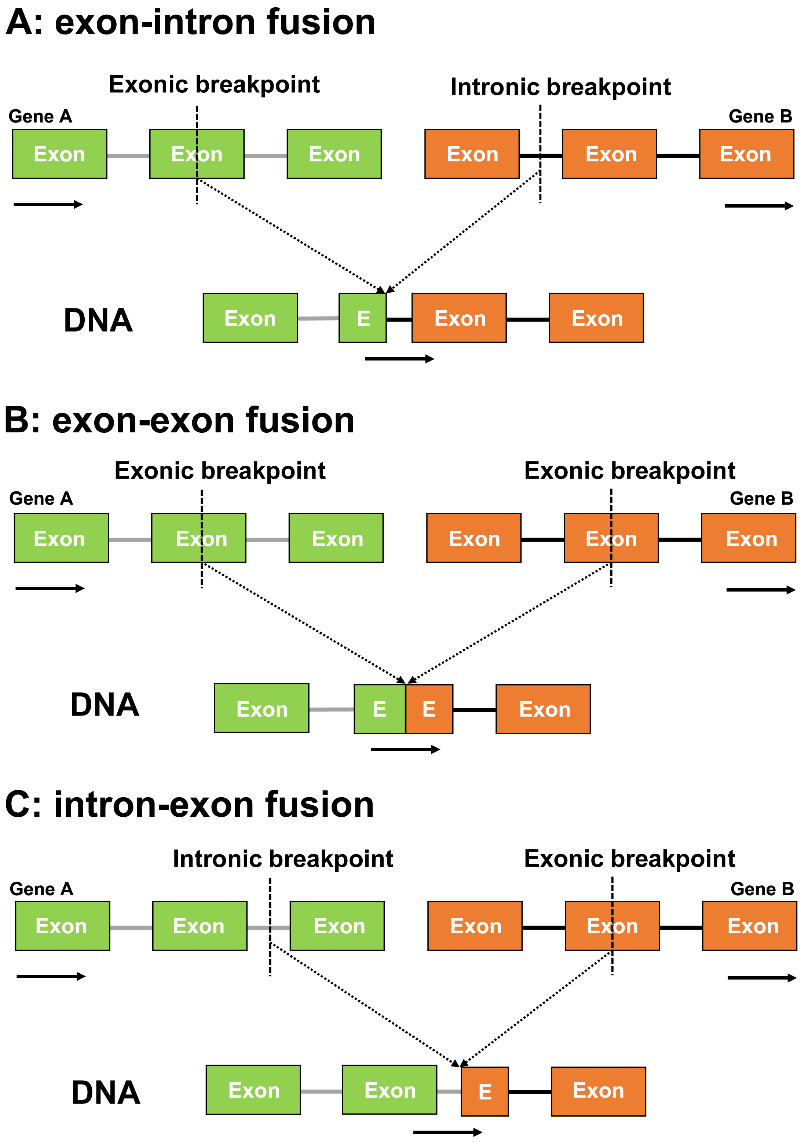


**Figure S1.** Schematic diagrams showing the formation of (A) “exon-intron”, (B) “exon-exon” and (C) “intron-exon” fusions at the DNA level. The rectangles indicate exons (E, exon), and the solid lines indicate introns. The arrows indicate the direction of transcription.


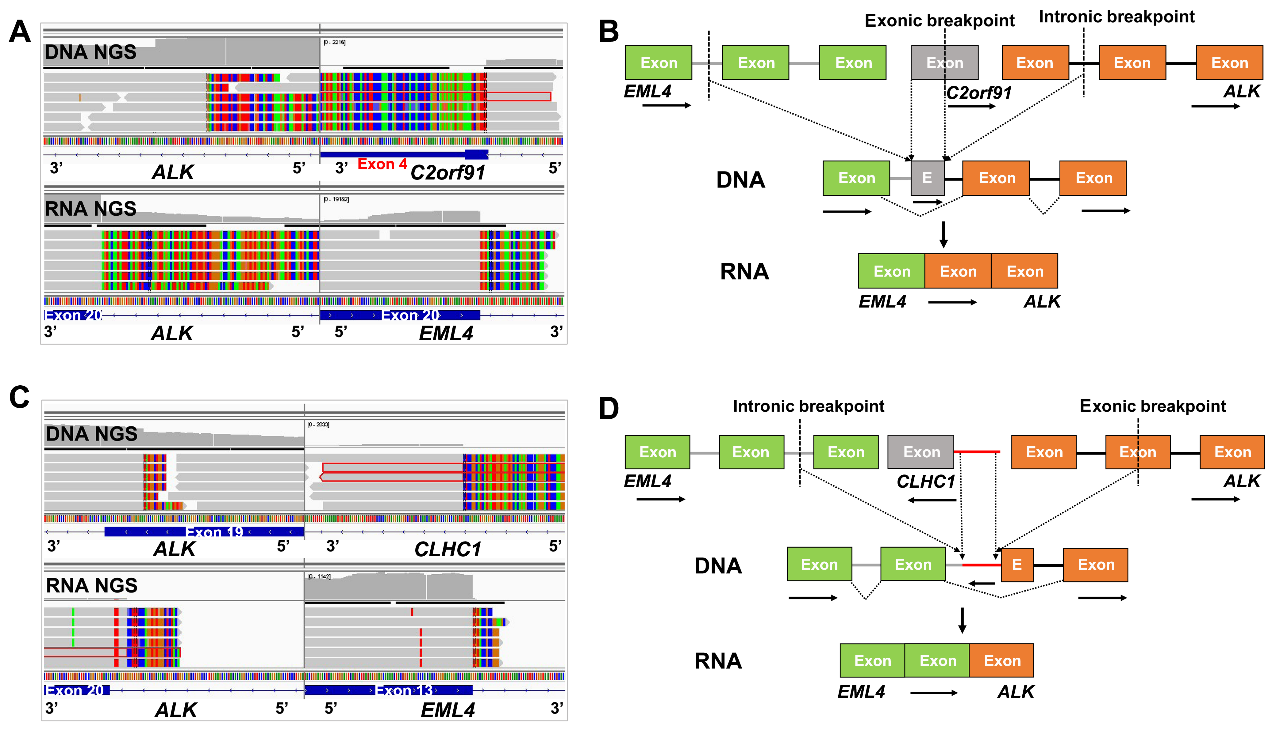


**Figure S2.** Examples and schematic diagrams of exonic-breakpoint fusions that generate functional transcripts with different partners. (A) In P35, DNA NGS showed an exonic-breakpoint *ALK* fusion involving *C2orf91* exon 4 and *ALK* intron 19, whereas RNA NGS detected an *ALK* fusion involving *EML4* exon 20 and *ALK* exon 20. (B) Schematic diagram of a possible mechanism leading to *C2orf91*-*ALK* detected by DNA NGS, but *EML4*-*ALK* detected by RNA NGS. (C) In P36, DNA NGS detected an exonic-breakpoint *ALK* fusion involving *CLHC1* intron 4 and *ALK* exon 19, whereas RNA NGS revealed an *ALK* fusion involving *EML4* exon 13 and *ALK* exon 20. (D) Schematic diagram of a possible mechanism leading to *CLHC1-ALK* detected by DNA NGS, but *EML4*-*ALK* detected by RNA NGS. The gray bars indicate sequencing reads that match the reference genome, and multicolored bars indicate mismatched reads (the corresponding partners). The rectangles indicate exons (E, exon), and the solid lines indicate introns. The arrows indicate the direction of transcription.
